# Nature-Inspired Hybrids (NIH) Improve Proteostasis by Activating Nrf2-Mediated Protective Pathways in Retinal Pigment Epithelial Cells

**DOI:** 10.3390/antiox11071385

**Published:** 2022-07-18

**Authors:** Ali Koskela, Federico Manai, Filippo Basagni, Mikko Liukkonen, Michela Rosini, Stefano Govoni, Massimo Dal Monte, Adrian Smedowski, Kai Kaarniranta, Marialaura Amadio

**Affiliations:** 1 Department of Ophthalmology, University of Eastern Finland, 70211 Kuopio, Finland; ali.koskela@uef.fi (A.K.); mikko.liukkonen@uef.fi (M.L.); kai.kaarniranta@uef.fi (K.K.); 2 Department of Biology and Biotechnology “L. Spallanzani”, University of Pavia, 27100 Pavia, Italy; federico.manai01@universitadipavia.it; 3 Department of Pharmacy and Biotechnology, University of Bologna, 40126 Bologna, Italy; filippo.basagni2@unibo.it (F.B.); michela.rosini@unibo.it (M.R.); 4 Department of Drug Sciences, Section of Pharmacology, University of Pavia, 27100 Pavia, Italy; govonis@unipv.it; 5 Department of Biology, University of Pisa, 56126 Pisa, Italy; massimo.dalmonte@unipi.it; 6 Interdepartmental Research Center Nutrafood “Nutraceuticals and Food for Health”, University of Pisa, 56124 Pisa, Italy; 7 Department of Physiology, Faculty of Medical Sciences in Katowice, Medical University of Silesia, 40752 Katowice, Poland; asmedowski@sum.edu.pl; 8 GlaucoTech Co., Ltd., 40752 Katowice, Poland; 9 Department of Ophthalmology, Kuopio University Hospital, 70211 Kuopio, Finland

**Keywords:** autophagy, age-related macular degeneration (AMD), cytoprotection, Nrf2, pharmacological modulation, SQSTM1/p62, oxidative stress, retinal pigment epithelium (RPE)

## Abstract

Antioxidant systems play key roles in many elderly diseases, including age-related macular degeneration (AMD). Oxidative stress, autophagy impairment and inflammation are well-described in AMD, especially in retinal pigment epithelial (RPE) cells. The master regulator of antioxidant defense Nrf2 has been linked to AMD, autophagy and inflammation. In this study, in human ARPE-19 cells, some nature-inspired hybrids (NIH1–3) previously shown to induce Nrf2-mediated protection against oxidative stress were further investigated for their potential against cellular stress caused by dysfunction of protein homeostasis. NIH1–3 compounds increased the expression of two Nrf2-target genes coding defense proteins, HO-1 and SQSTM1/p62, in turn exerting beneficial effects on intracellular redox balance without modification of the autophagy flux. NIH1–3 treatments predisposed ARPE-19 cells to a better response to following exposure to proteasome and autophagy inhibitors, as revealed by the increase in cell survival and decreased secretion of the pro-inflammatory IL-8 compared to NIH-untreated cells. Interestingly, NIH4 compound, through an Nrf2-independent pathway, also increased cell viability and decreased IL-8 secretion, although to a lesser extent than NIH1–3, suggesting that all NIHs are worthy of further investigation into their cytoprotective properties. This study confirms Nrf2 as a valuable pharmacological target in contexts characterized by oxidative stress, such as AMD.

## 1. Introduction

Age-related macular degeneration (AMD) is the most common reason for blindness among the elderly population in Western countries [1]. The disease affects the macular region of the eye responsible for sharp vision and leads to progressive worsening of the sight over the years. Thus, patients’ quality of life is compromised by the disease due to the loss of capacity to read, recognize faces and/or drive a car. AMD can be classified into two different forms, wet and dry, with 15% and 85% prevalence, respectively [1,2]. The dry form of the disease includes formation of protein–lipid plaques underneath the retina in the macular area. Conversely, in wet AMD, the clinical hallmark is the formation of new leaking blood vessels in the retina. Both forms of the disease can lead to death of retinal cells, which are responsible for vision [3]. Wet AMD can be treated with anti-VEGF (vascular endothelial growth factor) injections, slowing the disease’s progression, whereas dry AMD lacks effective treatment options [4,5].

In the retina, retinal pigment epithelial (RPE) cells are responsible for the homeostasis of photoreceptor cells, which convert light stimuli into electrical signals. Those signals are transferred to the visual pathway, comprised of neural retina and the optic nerve, which conveys the information to the brain [6]. Each RPE cell is connected to dozens of photoreceptors, providing nutrients and constituting the blood–retinal barrier between the retinal vasculature and outer retina [7]. RPE cells are quiescent, and therefore, cell proliferation is very restricted [8]. Thus, RPE cells’ functionality over the decades becomes an important factor when considering the health of the entire retina. However, RPE cells must be able to withstand one of the highest levels of oxidative stress met in the body due to their role in the retina [1]. Sources for reactive oxygen species (ROS) and nitrogen species (RNS) are many and comprise—but are not limited to—high cellular energy need and cellular metabolism, direct photo-oxidation and high polyunsaturated fatty acid content in phagocytosed photoreceptor outer segments (POS). Furthermore, many of the risk factors associated with AMD, such as aging, smoking, unhealthy diet and obesity, are linked to oxidative stress and undermine the functioning of antioxidant defense systems [9].

The antioxidant defense system has the ability to maintain the reduction–oxidation (redox) balance in the cell. Several factors participate in antioxidant defense system control: transcription factors and nuclear factors, with erythroid-2-related factor 2 (Nrf2) being the master regulator [10]. Nrf2 in its inactive state is bound to Kelch-like ECH-associated protein 1 (Keap1), which guides the ubiquitination and proteasomal degradation of Nrf2 [11,12]. Keap1 has cysteine residues capable of sensing oxidants or electrophilic molecules and changing the conformation of Keap1, leading to a release of Nrf2 and its translocation to the nucleus. In the nucleus, Nrf2 is guided to antioxidant response element (ARE) located at the promoter of several genes [13]. In fact, studies have shown hundreds of binding sites for Nrf2 in distinct pathways, even beyond the antioxidant defense system [14,15]. Dysfunctional Nrf2 regulation has been demonstrated to cause the development of AMD-resembling pathologies, and polymorphisms in the Nrf2 gene have been linked to AMD development, emphasizing the role of oxidative stress and the function of Nrf2 as the regulator of the antioxidant defense system [16,17,18].

When the antioxidant defense system’s ability to control oxidizing agents becomes overwhelmed, a condition termed oxidative stress—the oxidation of micro- and macromolecules within the cells—spreads rapidly, demanding efficient damage control to preserve cell homeostasis and survival. Both proteasomal degradation of damaged proteins and autophagy devoted to the digestion of damaged cell organelles and protein aggregates are important aspects of the whole antioxidant defense [19]. Autophagy can be categorized as one of the pathways activated by the same mechanisms as antioxidant defense systems [19]. In fact, the autophagy cargo receptor *SQSTM1*/*p62* (*sequestosome 1/p62*) gene has an ARE on its promoter region connecting the antioxidant defense system to autophagy [20]. SQSTM1/p62 is a classical selective autophagy receptor, but it also has roles in the ubiquitin–proteasome system, cellular metabolism, signaling and apoptosis. In autophagy, SQSTM1/p62 serves as cargo receptor, recognizing certain ubiquitination patterns of proteins, protein aggregates and cell organelles destined for autophagic clearance [21,22]. Furthermore, the formation of the autophagosome is thought to be guided by SQSTM1/p62-LC3 (Microtubule-associated protein 1A/1B-light chain 3) interaction, where autophagosomal membranes are built around SQSTM1/p62-tagged cargo based on recognition of LC3 [23]. During autophagosome maturation, the free cytosolic form of LC3 (LC3-I) becomes phosphatidylethanolamine (PE)-conjugated (LC3-II) and is bound to the membrane of the formed autophagosome, thus serving as a marker of autophagosome formation. In the final step, the autophagic cargo and autophagosomal membranes along with the SQSTM1/p62 and LC3-II, respectively, are degraded by the lysosomes after autophagosome-lysosome fusion. In addition to *SQSTM1/p62*, several other autophagy-related genes have been found to be controlled by Nrf2 [24].

The functionality of damage-limiting systems has been found to decline in AMD [25,26]. In fact, accumulation of ubiquitin and SQSTM1/p62 in the retinas of AMD patients and animal models of AMD are considered as signs of dysfunctional proteasomal and autophagic clearance, respectively [16,27]. Increased buildup of dysfunctional cell organelles and protein aggregates aggravates the oxidation status of the cell and may lead to inflammatory response and, ultimately, cell death. Inflammatory regulation can also be linked to antioxidant defense system activation via the Nrf2-nuclear factor kappa-light-chain-enhancer of activated B cells (NF-κB) crosstalk [28,29]. Additionally, heme oxygenase 1 (HO-1) and NAD(P)H quinone oxidoreductase 1 (NQO1), other downstream targets of Nrf2, can inhibit the production of proinflammatory mediators and suppress the expression of interleukin-6 (IL-6) and IL-8. IL-8, in particular, has been connected to the inflammatory process related to AMD [30].

**Figure 1:**
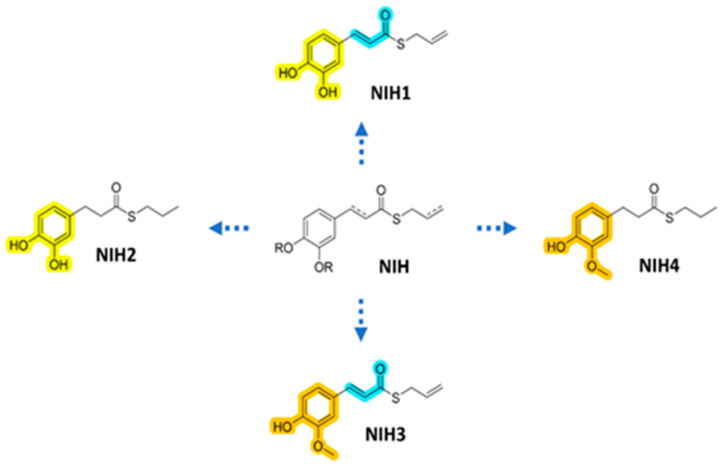
Molecular structures of NIH used in this study with different structural features highlighted: yellow for proelectrophilic catechol motif, blue for electrophilic α,β-unsatured carbonyl fragment and orange for curcumin-like o-methoxyphenol moiety.

Several nature-derived compounds have been found to activate Nrf2-dependent antioxidant defense [31]. Among their pleiotropic activities, electrophilic natural compounds have revealed the possibility of selectively modulating metabolic networks by targeting critical residues with properly tuned reactive moieties [32]. For example, the electrophilic modulation of redox regulatory proteins, such as Keap1, is a well-validated mechanism of action by which several Nrf2 inducers trigger Nrf2-mediated cytoprotective response [33]. In this context, a series of nature-inspired hybrids (NIH) joining hydroxycinnamic motif with mercaptan moiety revealed strong Nrf2-inducing properties in several biological systems [34,35,36,37,38]. In particular, these activities were restricted to compounds containing electrophilic (e.g., α,β-unsatured carbonyl) or proelectrophilic (e.g., catechol) fragments, suggesting nucleophilic addition of Keap1 cysteine residues to (pro)electrophilic portions of this set of compounds as the starting event of the pathway leading to gene expression. Since Nrf2 signaling and oxidative stress with damage-limiting autophagy and inflammatory processes are at the core of AMD and RPE cell pathology, a selection of NIH compounds in the ARPE-19 cell line (Figure 1) was studied, and the compounds provided valuable protection and prevention against oxidative-stress-induced damage [37]. Herein, we wanted to further investigate their involvement regarding the modulation of Nrf2 and related pathways, including autophagy and inflammation at the cellular level. 

## 2. Materials and Methods

### 2.1. NIH Syntheses and Characterization

NIH were synthesized and prepared according to previously published procedures [36,39]. Briefly, tert-butyldimethylsilyl protection of corresponding acid (e.g., caffeic acid for NIH1, ferulic acid for NIH3 and their saturated analogues for NIH2 and NIH4, respectively) followed by coupling with 2-propene-1-thiol (for obtaining NIH1 and NIH3) or 1-propanethiol (for NIH2 and NIH4) in the presence of *N*,*N*′-dicyclohexylcarbodiimide and 4-(*N*,*N*-dimethylamino) pyridine and a subsequent treatment with tetrabutylammonium fluoride gave compounds NIH1–4 an overall 19–45% yield. The detailed characterization of NIH1–4 by nuclear magnetic resonance (NMR) spectroscopy and electrospray ionization mass spectrometry (ESI-MS) was in agreement with those previously reported. NIH1–4 were determined >98% pure by HPLC analyses, which were carried out through HPLC reversed-phase conditions on a Phenomenex Jupiter C18 (150 × 4.6 mm I.D.) column (Phenomenex, Castel Maggiore, Italy), UV detection at λ = 302 nm (for NIH1 and NIH3) or 254 nm (for NIH2 and NIH4), a flow rate of 1 mL/min with mobile phase ACN/H_2_O 40:60 for NIH1, NIH3 and 50:50 for NIH2, NIH4. Analyses were performed on a liquid chromatograph model PU 2089 PLUS equipped with a 20 µL loop valve and linked to a MD 2010 Plus UV detector (Jasco Europe, Lecco, Italy).

### 2.2. Cell Culture and Treatments

The ARPE-19 cells used in the study were obtained from the American Type Culture Collection (ATCC, Manassas, VA, USA). The cells were grown in Dulbecco’s modified eagle medium/nutrition mix F12 (1:1, Gibco, Paisley, UK) supplemented with 10% fetal bovine serum (FBS, Hyclone, Logan, UT, USA), streptomycin (100 µg/mL, Lonza, Walkersville, MD, USA), Penicillin (100 U/mL, Lonza) and L-glutamine (2 mM, Lonza). The incubator settings were 37 °C for temperature and 5% CO_2_ in a humidified atmosphere. Cells were used in the study at passage numbers lower than 15.

Approximately 250,000 cells per well were seeded on 12-well plates and incubated for 48 h to reach confluence. The study compounds (NIH1–4) were introduced to the cells by removing the old culture medium and adding fresh medium containing 5 µM of each NIH compound diluted in dimethyl sulfoxide (DMSO) for a minimum of 3 h, according to our previous study on these compounds [37]. Proteasome inhibitor MG-132 (5 µM, Calbiochem, Billerica, MA, USA) and lysosome acidification inhibitor bafilomycin A1 (50 nM, MilliporeSigma, Burlington, MA, USA), both dissolved in DMSO, were added to the culture medium for 3–48 h after 24 h of NIH pretreatment time. Control cells were exposed to DMSO, the concentration of which was kept similar in all samples.

### 2.3. Protein Extraction and Western Blotting

After treatment, the cells were washed twice with Dulbecco’s phosphate-buffered saline (PBS, MilliporeSigma) and lysed in 75 µL of Mammalian Protein Extraction Reagent (M-PER, Thermo Fisher Scientific, Waltham, MA, USA). The M-PER reagent was left on the cells for 3 min, the wells were scraped on ice and the protein lysates were collected. Cytoplasmic fraction for Keap1 analysis was obtained by using the Nuclear Extract kit (Active Motif, Carlsbard, CA, USA) according to an established protocol [27]. The lysates were centrifuged at 13,000× *g* for 15 min at 4 °C and the supernatants were stored at −70 °C until analysis. The protein concentrations of ARPE-19 cell lysates were measured using the Bradford protein assay method. Samples containing 25 µg of protein were run into 15% SDS-PAGE gels. The protein bands were then transferred onto nitrocellulose membranes (GE Healthcare, Chicago, IL, USA) in an overnight wet blot. Ponceau S (MilliporeSigma) staining was performed on the membranes to confirm good protein transfer.

The membranes were cut in three parts, above the 70 kDa and 25 kDa bands, and blocked as follows: upper part in 5% BSA, 0.1% Tween^®^ 20 (MilliporeSigma) phosphate-buffered saline (T-PBS) solution; middle part in 3% milk, 0.3% T-PBS solution; lower part in 3% milk, 0.1% T-PBS solution, each for 1.5 h (upper and middle parts) or 2 h (lower part) at RT. The upper parts were then incubated overnight at +4 °C with Nrf2 primary antibodies (Novus biologicals LLC, Centennial, CO, USA) (1:1000 in 5% BSA, 0.1% T-PBS). The lower parts were incubated overnight at +4 °C with LC3 primary antibodies (Cell Signaling Technology, Danvers, MA, USA) (1:1000 in 5% BSA, 0.1% T-TBS). The middle parts of the membranes were incubated with primary antibodies for SQSTM1/p62 (Santa Cruz Biotechnology, Dallas, TX, USA) (1:1000 in 0.5% BSA, 0.3% T-PBS, overnight at +4 °C), HO-1 (Novus biologicals LLC), Keap1 (Novus Biologicals LLC), α-tubulin (MilliporeSigma) (1:8000 in 1% milk, 0.05% T-PBS; for 0.5 h at RT) or β-actin (Cell Signaling Technology) (1:6000 in 5% milk, 0.05% T-PBS; horseradish peroxidase-conjugated). After that, the membranes were washed for 3 × 5 min with their respective washing buffers. Horseradish peroxidase (HRP)-conjugated anti-mouse (MilliporeSigma) or anti-rabbit (Thermo Fisher Scientific) IgG secondary antibodies were then incubated at RT as follows: anti-mouse 1:10,000 in 3% milk, 0.3% T-PBS (SQSTM1/p62) for 2 h and anti-mouse 1:10,000 in 1% milk, 0.05% T-PBS (α-tubulin) for 0.5 h; or anti-rabbit 1:10,000 in 5% BSA, 0.1% T-PBS (Nrf2, HO-1) for 2 h; or anti-mouse 1:10,000 in 3% milk, 0.1% T-PBS (Keap1); or anti-rabbit 1:10,000 in 3% milk, 0.1% T-PBS (LC3) for 2 h. The membranes were then washed 3 × 5 min. Immobilon Western Chemiluminescent HRP Substrate (MilliporeSigma) was applied for 5 min, and the protein bands were detected using ImageQuant RT ECL Imager (GE Healthcare). The results were quantified using the ImageJ program (https://imagej.nih.gov/ij/, accessed on 30 April 2022).

### 2.4. RNA Extraction, Retrotranscription and Real-Time Quantitative PCR

Total RNA was extracted from ARPE-19 cells by the Direct-zol RNA MiniPrep Kit (Zymo Research, Irvine, CA, USA) and subjected to reverse transcription by the QuantiTect Reverse Transcription Kit (Qiagen, Hilden, Germany) following standard procedures. Real-time quantitative PCR (RT-qPCR) amplifications were carried out using the QuantiTect SYBR Green PCR Kit (Qiagen) and the Lightcycler instrument (Roche, Basel, Switzerland) with the following primers previously validated [40]: 

SQSTM1/p62 (Gene ID: 8878): 5′-CTGGGACTGAGAAGGCTCAC-3′ (upstream) and

5′-GCAGCTGATGGTTTGGAAAT-3′ (downstream);

GAPDH (Gene ID: 2597): 5′- CAGCAAGAGCACAAGAGGAAG-3′ (upstream) and

5′-CAACTGTGAGGAGGGGAGATT -3′ (downstream).

*GAPDH* mRNA was the reference to which all the values were normalized due to its substantial stability in our experimental conditions, as in most cases in literature. 2^−ΔΔCt^ method was used for the mRNA quantification.

### 2.5. Cell Viability Assay

ARPE-19 cells were plated 20,000/well in a 96-well plate, and cell viability was determined by PrestoBlue^®^ assay (Invitrogen) following manufacturer’s instruction. After treatments, cells were loaded for 30 min with PrestoBlue^®^ reagent prior to assay readout. Fluorescence was measured by the Synergy HT multidetection microplate reader (BioTek, Winooski, VT, USA) with excitation and emission wavelengths of 530 and 590 nm, respectively. The results are expressed as a percentage of the fluorescence of the samples in comparison to the control (100%).

### 2.6. ELISA

The cell culture medium from each treatment was collected and stored at −70 °C for IL-8 analysis. The OptEIA^TM^ IL-8 ELISA kit and the assay diluent were purchased from Becton Dickinson (Franklin Lakes, NJ, USA). Nunc 96-well plates (Thermo Fisher Scientific) were used, and the measurements were performed according to the instructions. The medium samples were thawed, diluted 1:32 and 1:64 (24 h and 48 h timepoints, respectively) and kept refrigerated until 15 min before pipetting onto the plate, at which point they were brought to RT. Each measurement plate had its own standard curve in triplicate and each sample in duplicate. Plates were washed with 0.05% Tween^®^ 20 PBS using a multichannel pipette. Once the substrate reactions had been stopped, the absorbances were measured using BioTek ELx808^TM^ Absorbance Reader (Winooski, VT, USA) at 450 nm, and the background absorbance at 562 nm was subtracted from the results.

### 2.7. Statistical Analyses

For the statistical analyses, the GraphPad InStat program (GraphPad 9.4.0. software, San Diego, CA, USA) was used. Data were subjected to analysis of variance (ANOVA) followed, when significant, by an appropriate post hoc comparison test, as specifically indicated. Differences were considered statistically significant when *p* < 0.05. All the experiments were performed using at least three independent biological replicas unless otherwise indicated.

## 3. Results

### 3.1. The Nrf2 Activators NIH1–3 Upregulate HO-1 and SQSTM1/p62 Protein Levels, Leading to a Decrease in Basal Autophagy Flux

**Figure 2:**
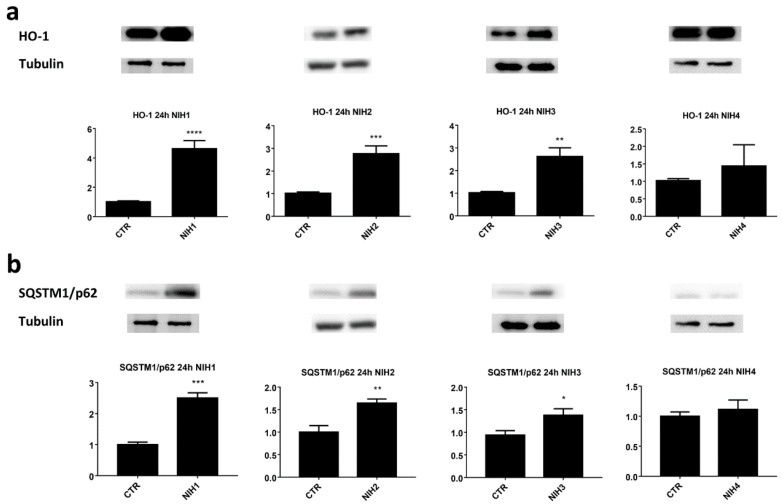
The effect of NIH compounds on the antioxidant defense system. ARPE-19 cells were treated with 5 µM of NIH compounds for 24 h, and the protein levels of (**a**) HO-1 and (**b**) SQSTM1/p62 were analyzed via Western blotting. The protein bands were quantified and normalized against α-tubulin. The results are expressed as fold change of the means compared to control (CTR) ± S.E.M. *n* = 6 for each experiment; * *p* < 0.05; ** *p* < 0.01; *** *p* < 0.001; **** *p <* 0.0001, paired *t*-test.

The levels of antioxidant defense-related proteins, Nrf2 and HO-1, were elevated after treatment with NIH1–3 compounds in ARPE-19 cells for 24 h (Supplementary Figure S1a, Figure 2a). In particular, NIH1 was able to increase the protein expression of both Nrf2 and HO-1, whereas NIH2 and NIH3 induced a statistically significant increase of the sole HO-1. Long-term exposure to NIH4 did not seem to influence the levels of the two antioxidant markers investigated, in agreement with previous findings showing lack of Nrf2 activation by short-term treatment with NIH4 in the same cell line [37]. Keap1 protein levels in the cytoplasmic fraction were analyzed in ARPE-19 treated with NIHs. As reported in Supplementary Figure S1b, a statistically significant decrease in Keap1 content was detected 3 h post-treatment with NIH1–3.

**Figure 3:**
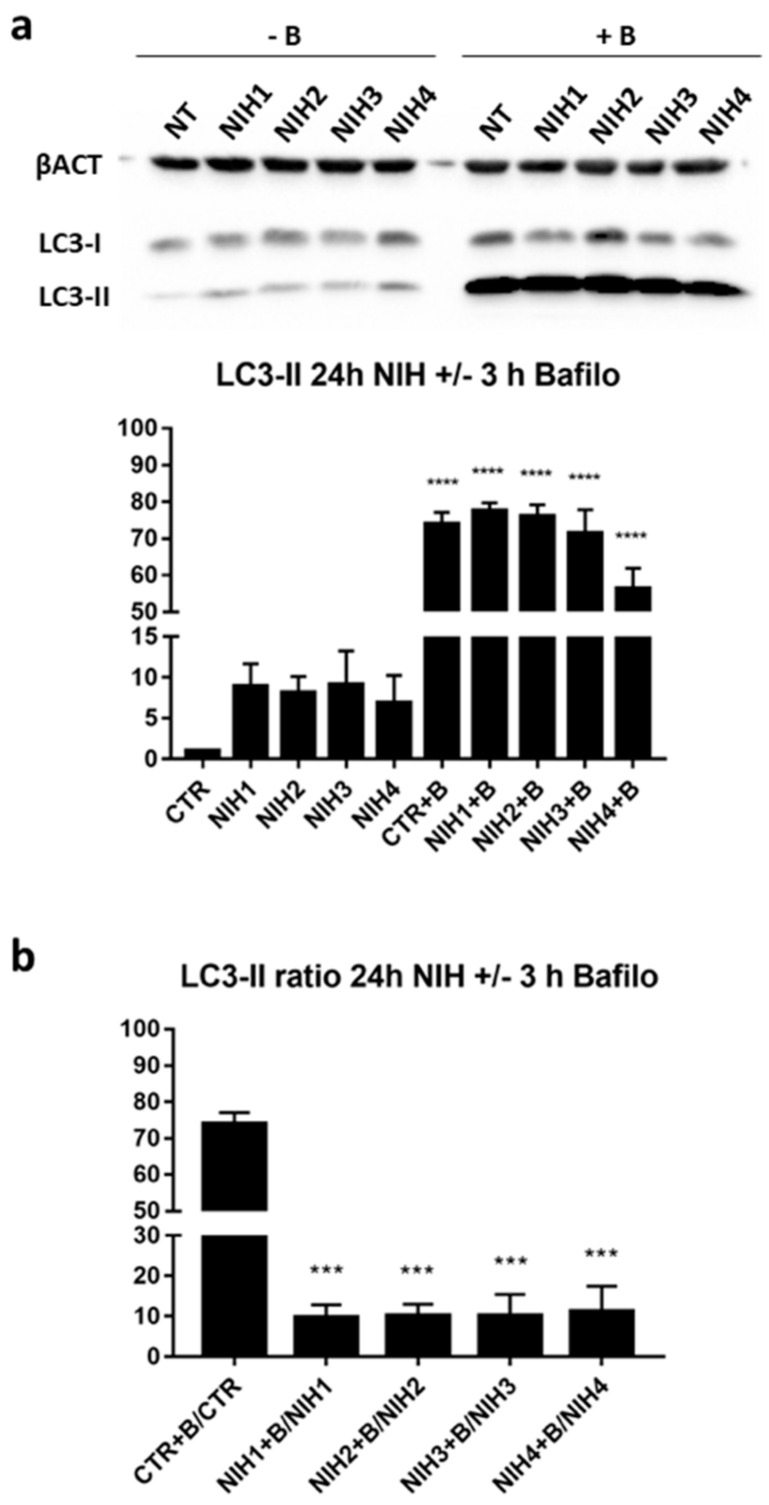
The effect of NIH compounds on autophagy flux upon lysosome acidification inhibitor bafilomycin A1 (B, 50 nM). ARPE-19 cells were treated for 24 h with NIH compounds before addition of bafilomycin A1 for 3 h. The protein levels of (**a**) LC3-II were analyzed via Western blotting. The protein bands were quantified and normalized against β-actin. The results are expressed as fold change of the means compared to control (CTR) ± S.E.M. *n* = 2; **** *p* < 0.0001, Dunnett’s multiple comparisons test. (**b**) The ratios of LC3-II protein levels between bafilomycin A1-treated ARPE-19 cells and relative controls are calculated (means ± S.E.M.). *n* = 2; *** *p* < 0.0005, Dunnett’s multiple comparisons test versus CTR + B/CTR.

Autophagy-related markers SQSTM1/p62 and LC3-II were also studied after NIH treatments. An increase in SQSTM1/p62 protein levels was observed after the treatment with the NIH1–3 compounds (Figure 2b). Considering the mutual influence of Nrf2 and SQSTM1/p62 on their own expression and their role in autophagy, with the aim to highlight possible NIH effects on autophagy flux, we evaluated the autophagy marker LC3-II levels in the presence or absence of bafilomycin A1, a lysosome acidification inhibitor used to halt the degradation of autophagosomes and thereby autophagy flux [41]. Bafilomycin A1 indeed gives an opportunity to assess autophagy direction (activation, inhibition or no effect) and rate by following the levels of LC3-II when its autophagy-mediated degradation is inhibited. Compared to untreated ARPE-19 cells, in basal conditions, NIHs increased LC3-II protein levels (although without any statistical significance) and—as expected—bafilomycin A1 treatment led to a further accumulation of LC3-II (Figure 3a). There were no differences between all the treatments in the bafilomycin-A1-treated group, indicating NIHs had no effect on autophagy flux, although the LC3-II ratio of each NIH plus bafilomycin A1 to the relative counterpart revealed an approximately eight-fold increase of LC3-II levels in comparison to the bafilomycin A1/control ratio (Figure 3b). 

**Figure 4:**
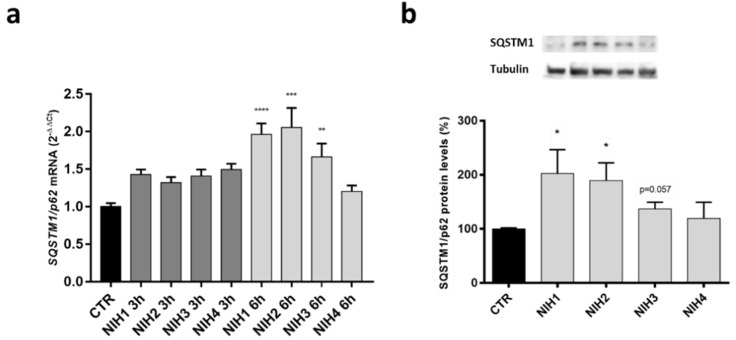
The effect of NIH compounds on the mRNA and protein expression of SQSTM1/p62. ARPE-19 cells were treated with 5 µM of NIH compounds for 3 h or 6 h. (**a**) The expression levels of *SQSTM1/p62* were measured with quantitative PCR after 3 h and 6 h treatment periods. The data were normalized against housekeeping gene *GAPDH,* and the results are shown as fold change compared to control ± S.E.M. *n* = 4. ** *p* < 0.01, *** *p* < 0.0005, **** *p* ≤ 0.0001, Dunn’s multiple comparisons test. (**b**) The protein levels of SQSTM1/p62 after NIH treatments for 6 h. The data were normalized against α-Tubulin (representative Western blotting gels are reported), and the results are shown as comparison to the control (CTR, 100%) ± S.E.M. *n* = 4. * *p* < 0.05, Dunn’s multiple comparisons test.

We then focused on Nrf2 downstream target *SQSTM1/p62*, which was further studied with quantitative PCR after 3 h and 6 h exposure to NIH compounds (Figure 4a). NIH1–3 significantly increased *SQSTM1/p62* mRNA expression after 6 h of treatment, whereas NIH4 showed no change in comparison to the control. The data from quantitative PCR are in line with the Western blot results obtained after 6 h of exposure, the protein levels of SQSTM1/p62 increased with NIH1 and NIH2, but not with NIH4 (Figure 4b). NIH3-induced increase of SQSTM1/p62 protein was on the borderline of statistical significance, suggesting that this compound requires longer times to upregulate SQSTM1/p62 protein, as observed for HO-1 protein in our previous study [37]. At 24 h, NIH1–3 compounds were able to increase the protein levels of HO-1 and SQSTM1/p62, known targets of Nrf2, indicating an activation of the antioxidant defense system.

**Figure 5:**
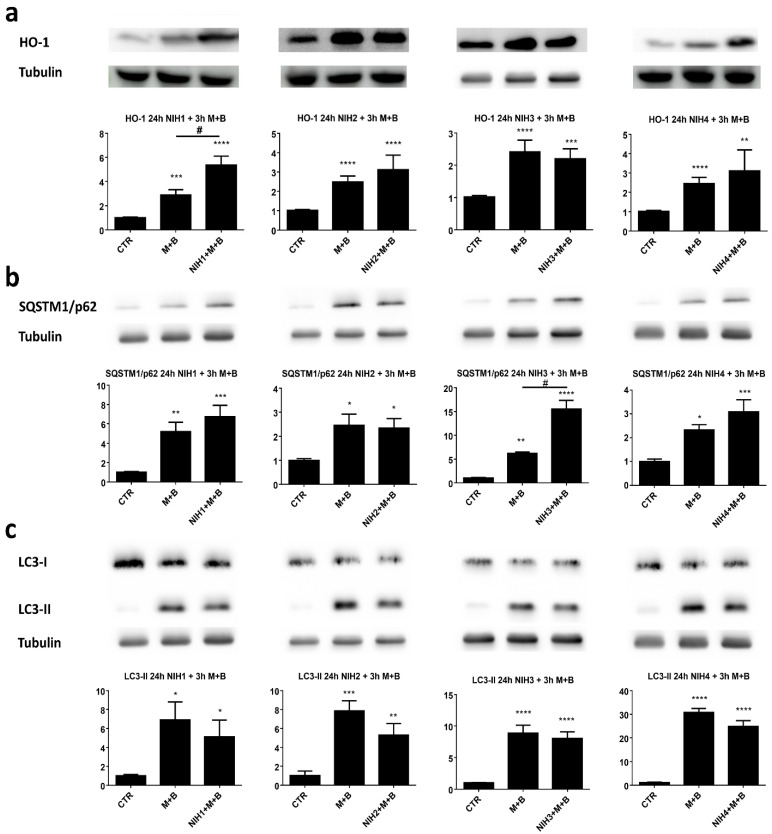
The effect of NIH compounds on the antioxidant defense system and autophagy upon inhibition of protein degradation systems. ARPE-19 cells were pretreated with NIH compounds for 24 h before addition of proteasome inhibitor MG-132 (M, 5 µM) and lysosome acidification inhibitor bafilomycin A1 (B, 50 nM) for 3 h. The protein levels of (**a**) HO-1, (**b**) SQSTM1/p62 and (**c**) LC3-II were analyzed via Western blotting. The protein bands were quantified and normalized against α-tubulin. The results are expressed as fold change of the means compared to control (CTR) ± S.E.M. *n* = 6, * *p* < 0.05, ** *p* < 0.01, *** *p* < 0.001, **** *p* < 0.0001, # = statistically significant difference between MG-132 + bafilomycin A1 (M + B) and M + B + NIH3 compound (NIH3 + M + B). Dunn’s multiple comparisons test for HO-1 and Dunnett’s multiple comparisons test—for SQSTM1/p62 and LC3-II, respectively—were used.

We then tested 24 h NIH1 pre-exposure in a model of massive dysfunction of protein homeostasis caused by a cotreatment with the proteasome inhibitors MG-132 and bafilomycin A1 for increasing durations. As expected, after 3 h, the stress stimulus alone increased the levels of all the proteins considered: Nrf2 (Supplementary Figure S1c), HO-1, SQSTM1/p62 and LC3-II (Figure 5). In addition, HO-1 levels were further increased by NIH1 pre-exposure in comparison with only MG-132 and bafilomycin A1 (Figure 5a). Likewise, NIH3 treatment resulted in significantly higher levels of SQSTM1/p62 protein upon the stress stimulus (Figure 5b). These findings encouraged us to continue the use of proteasome and autophagosome degradation inhibition for a longer time period.

### 3.2. NIH Compounds Protect Cellular Viability of ARPE-19 Cell upon Prolonged Proteasome and Autophagy Dysfunction

**Figure 6:**
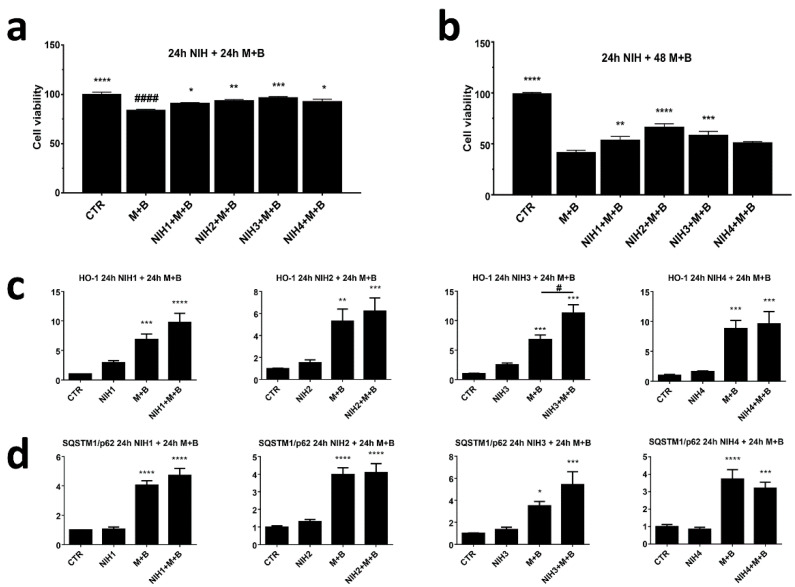
Cell viability and protein levels of HO-1 and SQSTM1/p62 of NIH-treated ARPE-19 cells upon inhibition of protein degradation systems. ARPE-19 cells were pretreated with NIH compounds for 24 h followed by proteasomal (M = MG-132, 5 µM) and lysosomal acidification (B = bafilomycin A1, 50 nM) inhibition for (**a**) 24 h or (**b**) 48 h. Cell viability was measured with PrestoBlue^®^, and the results are shown as percentage ± S.E.M. in comparison to control (CTR, 100%). The protein levels of (**c**) HO-1 and (**d**) SQSTM1/p62 were analyzed via Western blotting. The protein bands were quantified and normalized against α-tubulin. The results are expressed as fold change of the means compared to control (CTR) ± S.E.M. *n* = 6, * *p* < 0.05, ** *p* < 0.01, *** *p* < 0.001, **** *p* < 0.0001. # = statistically significant difference between MG-132 + bafilomycin A1 (M + B) and M + B + NIH1 compound (NIH1 + M+B) per Dunnett’s multiple comparisons test. #### = statistically significant difference between MG-132 + bafilomycin A1 (M + B) and CTR.

Prolonged protein aggregation with dysfunctional autophagic clearance leads to an oxidative-stress-prone cellular environment compromising cellular wellbeing. In fact, dysfunctional proteasome and autophagic activity decreased ARPE-19 cell viability after 24 h and 48 h exposure (Figure 6a,b). The viability of cells pre-exposed to NIH compounds for 24 h and then treated with stress stimulus for additional 24 h was instead comparable to that of the control (Figure 6a; Supplementary Figure S2). As expected, a longer treatment period with proteasome and autophagy inhibitors resulted in lower cell viability. Despite the poor condition of the cells after 48 h, cotreatment with NIH compounds 1–3 were able to significantly increase cell viability. NIH4, on the other hand, failed to influence cell survival after exposure to proteasome and autophagy inhibitors for 48 h.

Since there was a clear increase in cell viability with NIH1–3 compounds upon inhibition of protein degradation systems, protein levels of HO-1 and SQSTM1/p62 were analyzed after 24 h cotreatment with MG-132 and bafilomycin A1 (Figure 6c,d). Evaluation at 48 h showed that NIH1–3 kept the levels of HO-1 upregulated when added alone (fold-change versus CTR: 2.9 for NIH1; 1.5 for NIH2; 2.5 for NIH3; 1.5 for NIH4) (Figure 6c). Cotreatment with proteasome and autophagy inhibitors creating oxidative stress prone environment showed significantly more HO-1 when treated with NIH3. Overall, the levels of HO-1 kept rising from the 3 h time point upon proteasome and autophagy inhibition (Figure 5), suggesting long-term activation of antioxidant defense. The level of SQSTM1/p62 seemed to settle back to normal after 48 h when treated solely with NIH compounds (Figure 6d). The addition of MG-132 and bafilomycin A1 with or without NIH compounds caused significantly increased SQSTM1/p62 levels. Interestingly, higher SQSTM1/p62 protein levels than basal at 24 h seem to lead to higher HO-1 levels at later time points. This was especially evident with NIH3, for which the significant increase of SQSTM1/p62 seen after 3 h of proteasome and autophagy inhibition (Figure 5) resulted in a significant increase in the protein levels of HO-1 21 h later (Figure 6c). 

### 3.3. NIH Compounds Decrease IL-8 Secretion in Oxidative-Stress-Prone Environment Created by Dysfunctional Protein Clearance

**Figure 7:**
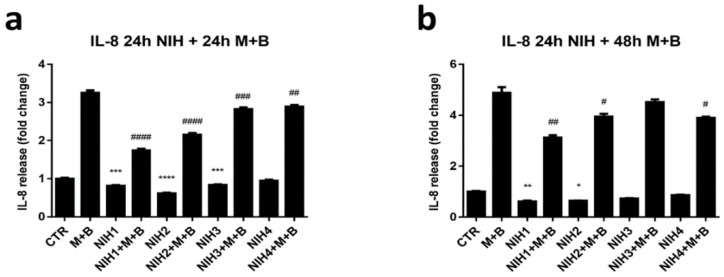
Effect of NIH compounds on IL-8 secretion with and without inhibition of protein degradation systems. ARPE-19 cells were pre-treated with NIHs for 24 h before addition of proteasome (M = MG-132, 5 µM) and autophagy (B = bafilomycin A1, 50 nM) inhibitors. The levels of IL-8 secretion were measured from cell culture media after (**a**) 24 h and (**b**) 48 h inhibition of protein degradative systems. The results are shown as fold-change of the mean in comparison to control (CTR) ± S.E.M. *n* = 4. * *p* < 0.05, ** *p* < 0.01, *** *p* < 0.001, **** *p* < 0.0001, Dunnett’s multiple comparisons test versus CTR (between CTR and NIH compound NIH1–4). # *p* < 0.05, ## *p* < 0.01, ### *p* < 0.001, #### *p* < 0.0001, Dunnett’s multiple comparisons test versus MG-132 + bafilomycin A1 (M + B) (between M + B and NIH1–4 + M + B).

Oxidative stress leads to inflammatory signaling and secretion of inflammatory markers. Protein aggregation model with dysfunctional proteasomal and autophagic protein clearance systems was used to determine the effect of NIH compounds on IL-8 secretion. ARPE-19 cells were pre-treated with NIH compounds for 24 h before addition of proteasome and autophagy inhibitors for 24 h and 48 h. NIH1–3 decreased the secretion of IL-8 when introduced to the cells solely for 48 h (Figure 7a). After 72 h the effect was maintained for NIH1 and 2, while it faded for NIH3 (Figure 7b). NIH4 did not influence IL-8 levels when added solely. When proteasome and autophagy inhibitors were introduced, the IL-8 decreasing effect of NIH compounds was evident after 24 h and 48 h exposure, with NIH1 showing the highest gap at both times, whilst the NIH3’s effect was almost abolished after 48 h. 

## 4. Discussion

The functionality of the antioxidant defense system has an important role in highly stressed tissues and cells such as the retina in the eye. A dysfunctional or inadequate antioxidant defense system leads to the accumulation of damaged cellular organelles and proteins, worsening the oxidative stress met by the cells [42]. Long-term effects may lead to a persistent inflammatory response and, eventually, cell death. Therefore, securing the function of the antioxidant defense system becomes one of the cornerstones in therapeutical or preventative strategies treating diseases with high oxidative stress, such as AMD.

NIH compounds 1–3 clearly activated the antioxidant defense system, as seen from increased levels of Nrf2 and decreased levels of Keap1 (Supplementary Figure S1a,b)—as well as increased expression levels of its known targets, HO-1 and SQSTM1/p62 (Figure 2)—although the effect on cellular Nrf2 levels was statistically significant only with NIH1. However, the activation of the antioxidant defense system by Nrf2 is regulated by its dissociation from its inhibitory partner, Keap1, and translocation to the nucleus rather than the upregulation of its expression [43]. The redox balance in the cell is important for normal biological functions; Nrf2’s dissociation from Keap1 can also drive Nrf2 to degradation via proteasome in basal conditions. In fact, short-term proteasome inhibition resulted in a significant increase in cellular Nrf2 levels (Supplementary Figure S1c), emphasizing the importance of proteasomal clearance on Nrf2 turnover. At the same time, NIH compounds were not able to further increase the levels of Nrf2, suggesting an activation mechanism involving translocation of Nrf2 from the cytosol to the nucleus rather than an actual increase in its expression levels.

High oxidative stress creates a demand for efficient removal of damaged proteins and cell organelles, which are degraded and recycled via proteasomal and autophagic processes [42]. Autophagy is triggered when proteins start to aggregate or proteasomal degradation is insufficient [44]. As is well known, this condition is further enhanced by an increase in ROS, which are physiologically produced during the phototransduction process in the RPE and surrounding cells. Based on these premises, autophagy flux was investigated after the treatment with the NIH compounds in the presence/absence of bafilomycin A1. This revealed no changes in the degradative pathway, as attested by LC3-II protein levels being comparable among all treatments in the bafilomycin A1 group (Figure 3a). However, when comparing each pair of NIH with/without bafilomycin A1 and bafilomycin A1/control, the LC3-II ratios of the former are much lower (Figure 3b). These findings suggest that in basal conditions without bafilomycin-1, NIH-treated ARPE-19 cells required a slower autophagy than controls due to the beneficial effects exerted by NIHs on proteostasis and intracellular redox homeostasis, likely through the induction of Nrf2, HO-1 and SQSTM1/p62. From the reverse perspective, we may infer that ARPE-19 cells require a faster autophagy rate than NIH-treated cells. This intriguing hypothesis is supported by our previous study of ARPE-19 cells exposed to oxidative stress stimulus, which showed that all NIH1–4—due to their molecular structure—are endowed with direct antioxidant effects, with NIH1 and NIH4 exerting the strongest and lowest reducing activity, respectively [37].

The present findings indicate that NIH compounds did not influence autophagy upon impaired proteostasis, pointing to a distinct regulation between Nrf2-driven antioxidant defense systems and autophagy. This was further confirmed by using autophagy inhibitor bafilomycin A1 and proteasome inhibitor MG-132 to induce cellular stress by mimicking massive dysfunctional protein degradation induced by the blockage of both the proteasome and autophagic pathways; despite this stress, LC3-II levels were comparable between NIH-treated and untreated cells (Figure 5d).

The increase in SQSTM1/p62 protein content in cells treated with NIH1–3 compounds (Figure 2b) could be explained by the binding site for Nrf2 (ARE) at the promoter region of the *SQSTM1/p62* gene [20], in agreement with the upregulation of *SQSTM1/p62* mRNA following short-term exposure of ARPE-19 cells to NIH compounds 1–3 (Figure 4). It is generally accepted that SQSTM1/p62 can create a positive feedback loop leading to enhanced activation of Nrf2 (non-canonical activation of Nrf2) [45]. In particular, SQSTM1/p62 can be upregulated by Nrf2, and it competes with Nrf2 for the same binding site in Keap1, leading to increased levels of unbound Nrf2, with the potential to further activate the antioxidant defense system. Interestingly, in our system, higher SQSTM1/p62 levels correlate with higher HO-1 levels upon NIH treatments with or without proteasome and autophagy inhibition (Figure 2 and Figure 5). For example, NIH1 and NIH2 treatments increased SQSTM1/p62 levels up to 2.5-fold and 1.6-fold and HO-1 levels 4.8-fold and 2.7-fold, respectively (Figure 2). Similarly, NIH3 significantly increased SQSTM1/p62 levels after 3 h of proteasome and autophagy inhibition, and the same effect was seen on HO-1 levels at the later time point (Figure 5 and Figure 6c,d). Nrf2 activation by the NIH treatment or proteasome/autophagy inhibition also led to SQSTM1/p62-orchestrated positive feedback loop and further enhanced the antioxidant defense system. In our experiments, we did not detect increased levels of Nrf2, HO-1 and SQSTM1/p62, except for NIH1 and NIH3. This encourages future experiments with submaximal doses of MG-132 and bafilomycin A1 or other stressors to evaluate whether NIH compounds can, in the longer term, further increase the amount of Nrf2 and/or its targets. Moreover, in vivo studies evaluating NIHs’ effects in the retina of AMD animal models characterized by Nrf2 impairment will be of interest to determine the potential translatability of our bench results to the pre-clinical phase. 

According to literature, as a result of oxidative stress, inflammatory processes are activated [46,47]. Inflammation can be considered one of the core elements in oxidative-stress-related diseases. All NIH compounds were able to attenuate IL-8 secretion upon proteasome and autophagy inhibition after 24 h in ARPE-19 cells (Figure 7a). The anti-inflammatory effect of NIH3 was substantially lost after 48 h (Figure 7b). Similarly, all NIH compounds protected cells from death after 24 h of proteasome and autophagy inhibition (Figure 6a). The cytoprotective effect of NIH4 faded after prolonged inhibition of protein degradation systems (Figure 6b). However, NIH4 did not seem to influence the antioxidant defense system (Figure 2 and Figure 4), suggesting that its anti-inflammatory effect involves other pathways rather than controlling oxidative stress via Nrf2. Considering the chemical structures of NIH compounds, NIH1 and NIH2 carry a catechol moiety with/without an α,β-unsaturated carbonyl group, respectively, similarly to classical Nrf2 activators such as quercetin [48]. NIH3 and NIH4 carry a methoxyphenol ring with/without an α,β-unsaturated carbonyl group, respectively, and correspond more to curcumin structurally [49]— especially NIH3, which possesses both. However, curcumin has been proposed to activate Nrf2 via several pathways, including PKCδ-mediated phosphorylation of SQSTM1/p62 and Keap1 histone modification [50,51]. Furthermore, curcumin has been shown to activate an anti-inflammatory response via the TNF-α/NF-κB pathway, and Nrf2 has been proposed to be one controller of the inflammation-related pathways [28,52,53]. Thus, in ARPE-19 cells, NIH1–2 compounds may have positive effects on inflammation mainly by controlling oxidative stress and activating Nrf2, whereas NIH3–4 may also stimulate anti-inflammatory pathways Nrf2-independently. This hypothesis is in agreement with previous findings in another human cell line [47] and in ex vivo mouse retinal explants [38]. It is interesting to point out that none of the NIH compounds 1–4 affected IL-8 release from a human monocyte cell line under LPS exposure [47], suggesting that the effects of NIH on IL-8 secretion may vary between cell types and/or be cell-specific. NIH compounds with dual effects on these key pathways are thus worthy of further investigation. In particular, future experiments will be aimed at clarifying the mechanism through which NIHs induce a decrease in IL-8 release. 

Importantly, in ex vivo mouse retinal explants exposed to oxidative stress stimulus, six days incubation with NIH1 induced a massive and persistent expression of antioxidant enzymes via Nrf2 pathway activation, preventing the accumulation of ROS, retinal cell apoptosis and glial reactivity [38].

## 5. Conclusions

**Figure 8:**
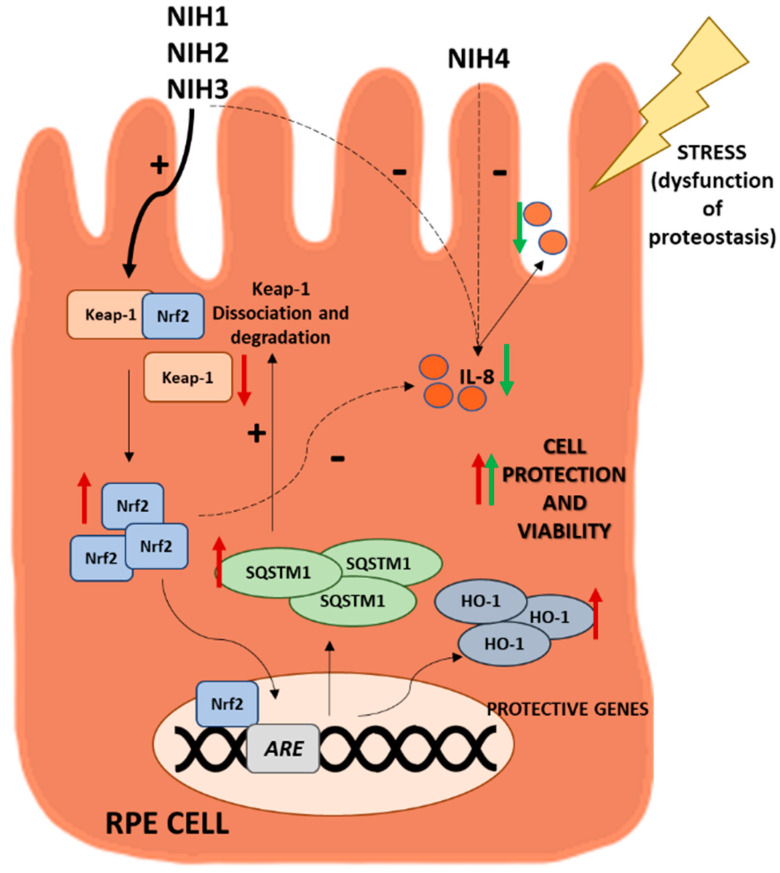
Summary of the main protective effects induced by treatment with NIH compounds in ARPE-19 cells in normal conditions and under inhibitors of protein degradation systems. NIH1–3 are able to activate Nrf2 by binding to Keap1 proteins, inducing a decrease in them. Nrf2 induces upregulation of both HO-1 [37] and SQSTM1/p62. Moreover, these compounds decrease IL-8 release and improve cell viability under stress. NIH4 shows a mild effect on cell survival and IL-8 release via an Nrf2-independent pathway. See the text for details.

In conclusion, in ARPE-19 cells, Nrf2 pathway activation by NIHs limited oxidative stress, reduced inflammation and enhanced cell viability (Figure 8) by likely ameliorating proteostasis, further confirming Nrf2 as a valuable pharmacological target in contexts characterized by oxidative stress. 

## Data Availability

All of the data is contained within the article and the Supplementary Materials.

## Supplementary Materials

The following supporting information can be downloaded at: https://www.mdpi.com/article/10.3390/antiox11071385/s1, Figure S1: Total Nrf2 and cytoplasmic Keap1 protein levels following NIH compounds exposure in ARPE-19 cells; Figure S2: Microscopical images of ARPE-19 cells pre-treated with NIH1 for 24 h before the addition of proteasome and autophagy inhibitors.
