# Supplementary material for: Nature-Inspired Hybrids (NIH) Improve Proteostasis by Activating Nrf2-Mediated Protective Pathways in Retinal Pigment Epithelial Cells
Source: Antioxidants (Basel). 2022 Jul 18;11(7):1385. doi: 10.3390/antiox11071385 (PMC9312215; doi:10.3390/antiox11071385)
Supplement: Supplementary file 1 [file antioxidants-11-01385-s001.zip › antioxidants-1770796-supplementary-update.pdf]

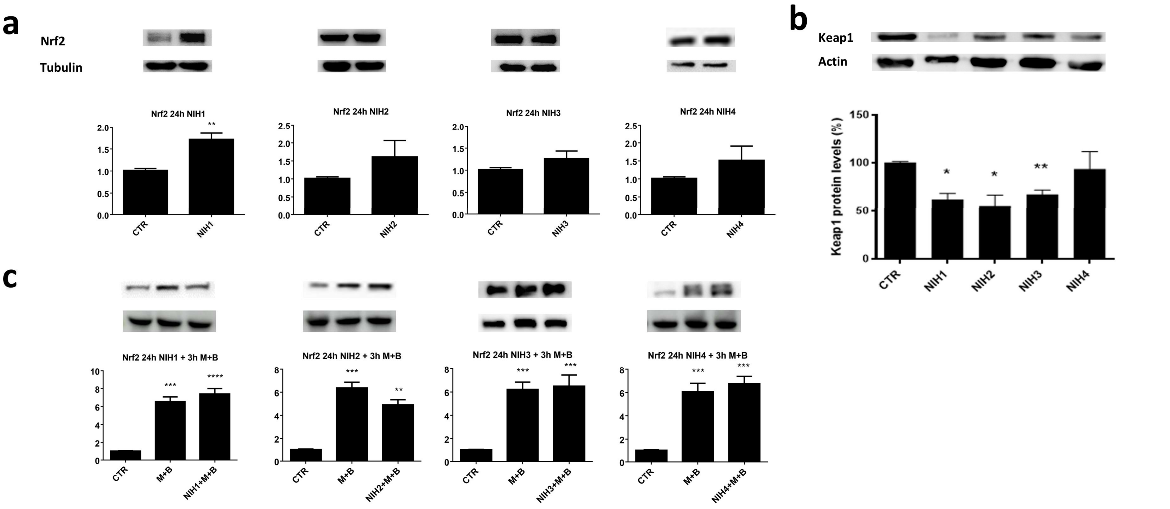

Supplementary Figure S1. Total Nrf2 and cytoplasmic Keap1 protein levels following NIH compounds exposure in ARPE-19 cells. ARPE-19 cells were treated with 5 μM of NIH compounds for the times reported in figures and the protein levels of (a, c) Nrf2, and (b) Keap1 were analyzed via Western blotting. The protein bands were quantified and normalized against α-tubulin or β-actin. The results are expressed as fold-change of the means compared to control (CTR) ± S.E.M. $n = 6$ for each experiment, ** = $p < 0.01$, Paired t-test (panel a); * = $p < 0.05$, ** = $p < 0.01$, Welch ANOVA (panel b); ** = $p < 0.01$, *** = $p < 0.001$, **** = $p < 0.0001$, Dunn's multiple comparisons test (panel c).

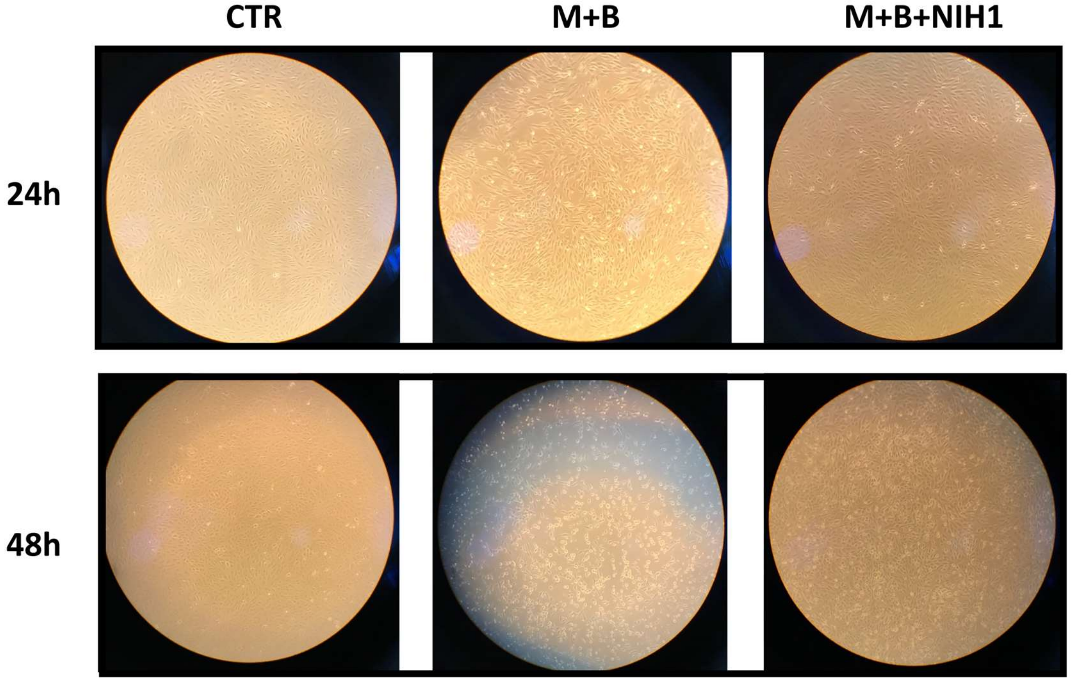

Supplementary Figure S2. Microscopical images of ARPE-19 cells pre-treated with NIH1 (5 μM) for 24 h before addition of proteasome (M = MG-132, 5 μM) and autophagy (B = Bafilomycin A1, 50 nM) inhibitors for 24 h (upper) and 48 h (lower). The images of M+B+NIH1 are representative also of the effects of Nrf2-activators NIH2 and NIH3.
